# A bivalent subunit vaccine efficiently produced in *Pichia pastoris* against SARS-CoV-2 and emerging variants

**DOI:** 10.3389/fmicb.2022.1093080

**Published:** 2023-01-10

**Authors:** Huifang Xu, Tiantian Wang, Peng Sun, Xuchen Hou, Xin Gong, Bin Zhang, Jun Wu, Bo Liu

**Affiliations:** Department of Microorganism Engineering, Beijing Institute of Biotechnology, Beijing, China

**Keywords:** SARS-CoV-2, RBD, subunit vaccine, bivalent vaccine, glycoengineered yeast

## Abstract

The emergence of severe acute respiratory syndrome coronavirus type II (SARS-CoV-2) variants have led to a decline in the protection of existing vaccines and antibodies, and there is an urgent need for a broad-spectrum vaccination strategy to reduce the pressure on the prevention and control of the pandemic. In this study, the receptor binding domain (RBD) of the SARS-CoV-2 Beta variant was successfully expressed through a glycoengineered yeast platform. To pursue a more broad-spectrum vaccination strategy, RBD-Beta and RBD-wild type were mixed at the ratio of 1:1 with Al(OH)_3_ and CpG double adjuvants for the immunization of BALB/c mice. This bivalent vaccine stimulated robust conjugated antibody titers and a broader spectrum of neutralizing antibody titers. These results suggested that a bivalent vaccine of RBD-Beta and RBD-wild type could be a possible broad-spectrum vaccination strategy.

## 1. Introduction

Coronavirus disease 2019 (COVID-19) is a highly infectious disease caused by severe acute respiratory syndrome coronavirus type II (SARS-CoV-2), which to date has globally caused over six billion infections and over six million deaths,^1^ posing a huge threat to public health security. SARS-CoV-2 contains a trimeric surface spike protein (S), which consists of an S1 subunit that binds to the host cell and an S2 subunit that fuses to the cell membrane (Gstöttner et al., 2021). The S2 subunit mediates membrane fusion, allowing the virus to invade the host cell (Lan et al., 2020), thereby enabling infection of the human body and triggering a series of changes in bodily functions (Harrison et al., 2020). The wide range of hosts and the coronavirus genomic structure make it susceptible to genetic mutations and recombination, and consequently they exhibit a rich genetic diversity, enabling several SARS-CoV-2 variants to escape immunity and become difficult to inhibit even using vaccines (Harvey et al., 2021; Hoffmann et al., 2021; Souza et al., 2022; Zhang et al., 2022). The World Health Organization classifies variants into two categories, variants of concern (VOCs) and variants of interest (VOIs), with VOCs being more infectious and drug-resistant than VOIs are. Delta and Omicron, which emerged in November 2021 with a large number of mutated sites and a rapid increase in cases, are all VOCs. Current vaccines are not sufficiently protective against Omicron (Planas et al., 2022). In particular, vaccine protection against the BA.4 and BA.5 variants was significantly decreased (Tuekprakhon et al., 2022), and it is difficult for vaccine development to maintain pace with viral antigenic change.

The receptor binding domain (RBD) is considered an ideal antigen for vaccine design because of the predominantly neutralizing epitopes (Kuo et al., 2020; Arunachalam et al., 2021). Three of the mutation sites located in the RBD region of Beta variant, K417N, E484K, and N501Y, alter the virus transmission capacity (Tegally et al., 2021; Yuan et al., 2021) and can cause immune escape (Moyo-Gwete et al., 2021), resulting in reduced protection of vaccines (Wang et al., 2021; Cameroni et al., 2022). It is therefore worthwhile to consider the development of multivalent vaccines.

Our laboratory previously knocked out the excessive mannosylation modification pathway of the yeast expression system and reconstructed the mammalian glycosylation modification pathway based on yeast glycosyl engineering. Based on this platform, we used glycoengineered yeast to rapidly and efficiently express recombinant RBD of SARS-CoV-2 (Liu et al., 2022). Here, we developed a specific vaccine against SARS-CoV-2 Beta variant. During this research, we found that the sera of RBD-Beta vaccine-immunized mice had a significantly reduced neutralizing activity against SARS-CoV-2 wild type (WT) pseudovirus. Consequently, we prepared a bivalent vaccine using the SARS-CoV-2 RBD-WT and RBD-Beta with Al(OH)_3_ and CpG double adjuvants for immunization of BALB/c mice. The results showed that the bivalent vaccine exhibited a degree of broad-spectrum neutralizing activity.

## 2. Materials and methods

### 2.1. *Pichia pastoris*, bacterial strains, and materials

The method used for constructing glycoengineered *Pichia pastoris* has been described previously (Choi et al., 2003). *P. pastoris* was cultivated in yeast peptone dextrose medium at 25°C. *Escherichia coli* Trans5α (TransGen Biotech, Beijing, China) was cultured in Luria-Bertani medium at 37°C. Yeast extract, agar, and tryptone were obtained from OXOID (Basingstoke, United Kingdom); NaCl was purchased from SINOPHARM (Shanghai, China). Hygromycin B, zeocin, and G418 were obtained from Thermo Fisher (MA, United States).

### 2.2. RBD-Beta protein expression, fermentation, and purification

The SARS-CoV-2 RBD gene (GenBank accession number MN908947.3) was synthesized, ligated into the pPICZαA vector, and subsequently transferred into glycoengineered *P. Pastoris* cells and RBD-wild type protein (RBD-WT) was successfully expressed and purified (Liu et al., 2022). The gene sequence of K417N, E484K, and N501Y sites were mutated, and the target gene was cloned to generate expression vector pPICZαA-RBD-Beta. BglII-linearized pPICZαA-RBD-Beta was transferred into glycoengineered *P. pastoris* cells by electric shock, and expression of the target protein was induced by addition of methanol. Positive clones were screened by SDS-PAGE and western blot. The primary antibody was anti-RBD-WT rabbit serum (produced and kept in our laboratory) and the secondary antibody was goat anti-rabbit-HRP (1:2500, SAB3700885, SIGMA).

The positive strain highly expressing RBD-Beta was fermented in a 5 L biofermenter, and the target protein was induced with methanol for expression. The supernatant was passed through the following chromatographic columns: Capto MMC, Phenyl Sepharose Fast Flow, Source 30Q, Source 30S, and Superdex-G75 (all from GE Healthcare, Cal., United States) to obtain high purity RBD-Beta protein.

### 2.3. Glycosylation validation and purity of RBD-Beta

The purified RBD-Beta protein was digested with peptide-N-asparagine amidase F (PNGF) and incubated at 37°C for 12 h. After digestion, western blotting was performed following 12% SDS-PAGE.

The purity of RBD-Beta was tested using size exclusion chromatography-high performance liquid chromatography (SEC-HPLC) and reversed phase-high performance liquid chromatography (RP-HPLC), and absorbance values at 280 nm were recorded (Agilent 1260 HPLC).

### 2.4. RBD-Beta and RBD-WT binding capacity to hACE2-his

RBD-Beta and RBD-WT were diluted to 4 μg/ml with coating solution (pH 9.6, 50 mmol/L carbonate) and added 100 μl to each well of a 96-well ELISA plate. The plate was incubated overnight at 4°C. Phosphate buffered saline (PBS) containing 0.1% Tween20 (PBST) was used to wash the plate twice, and then each well was incubated with 300 μl of 5% skimmed milk at 37°C for 1 h. The milk was discarded, and the plate was washed three times with PBST. His-tagged human angiotensin-converting enzyme 2 (hACE2-His, produced and kept in our laboratory, 200 μl at 8 μg/ml) diluted with PBST was added to the first well and then 100 μl was used to create an 11-step dilution series across the plate. After incubation at 37°C for 1 h, 100 μl of anti-His antibody (SIGMA) was added to each well and incubation was continued at 37°C for 1 h. The plate was then washed four times with PBST and developed with 100 μl/well of TMB one-component chromogenic solution for 4 min; the reaction was stopped with 50 μl/well of 2 M H_2_SO_4_. The results were detected with a microplate reader at 450 nm.

### 2.5. RBD-Beta and bivalent vaccine formulation and immunization

Female BALB/c mice 6–8 weeks old (Beijing Weitonglihua Laboratory Animal Technology Co., Ltd.) were acquired and kept in the Animal Center of Beijing Institute of Biotechnology. The experimental animal welfare ethics number was IACUC-DWZX-2020-039.

For testing the immunogenicity of RBD-Beta vaccine, mice were randomly divided into the following six immunization groups: 10 μg RBD-WT/50 μg CpG/100 μg Al(OH)_3_ (*n* = 5); 5 μg RBD-Beta/50 μg CpG/100 μg Al(OH)_3_ (*n* = 5); 10 μg RBD-Beta/50 μg CpG/100 μg Al(OH)_3_ (*n* = 5); 10 μg RBD-Beta/100 μg Al(OH)_3_ (*n* = 5); 50 μg CpG/100 μg Al(OH)_3_ (AD; *n* = 5); and normal saline (NS) group (*n* = 5).

For the detection of bivalent vaccine, mice were randomly divided into the following seven immunization groups: 2.5 μg RBD-WT + 2.5 μg RBD-Beta/50 μg CpG/100 μg Al(OH)_3_ (*n* = 10); 5 μg RBD-WT + 5 μg RBD-Beta/50 μg CpG/100 μg Al(OH)_3_ (*n* = 10); 10 μg RBD-WT/50 μg CpG/100 μg Al(OH)_3_ (*n* = 10); 10 μg RBD-Beta/50 μg CpG/100 μg Al(OH)_3_ (*n* = 10); 2.5 μg RBD-WT + 2.5 μg RBD-Beta/100 μg Al(OH)_3_ (*n* = 10); 5 μg RBD-WT + 5 μg RBD-Beta/100 μg Al(OH)_3_ (*n* = 10); and 50 μg CpG/100 μg Al(OH)_3_ adjuvant groups (AD; *n* = 10).

Vaccines were prepared by adding CpG and Al(OH)_3_ double adjuvants at room temperature while stirring and diluting to the corresponding concentration with saline. For bivalent vaccine, RBD-WT and RBD-Beta were mixed at the ratio of 1:1. Mice were immunized twice on days 0 and 14 with 100 μl of immunization in the hind leg of each mouse. Blood was collected before immunization on days 0, 14, and 28, and spleen cells from each of the seven groups were taken for enzyme-linked immunospot (ELISpot) testing on day 10 after the second immunization.

### 2.6. ELISA for conjugated antibody titers of vaccines in immunized mice

Blood was collected from post-immunized animals on days 14 and 28 after the first immunization. After blood collection, supernatant was collected by centrifugation at 10,000 rpm for 8 min at 4°C, and stored at −80°C. RBD-WT and RBD-Beta were then diluted to 2 μg/ml using coating buffer, and 100 μl was added, respectively, to each well of the ELISA plates and the plates were placed at 4°C overnight. The plates were then washed twice with PBST, 300 μl of 5% skimmed milk was added per well; incubation was continued for 1 h at 37°C. Mouse serum at 1:10,000-fold dilution was added to the first well and then diluted 11 times (including the first well), with two replicate wells per sample. The plates were then incubated at 37°C for 1 h and were washed three times with PBST. After 1 h incubation at 37°C, 100 μl of goat anti-mouse IgG (HRP) conjugated antibody (1:5,000 dilution) per well was added and the wells were incubated for 1 h at 37°C. Plates were then washed four times with PBST and developed with 100 μl/well of TMB one-component chromogenic solution for 4 min; 50 μl/well of 2 M H_2_SO_4_ was added to terminate the reaction. The results were detected with a microplate reader at 450 nm.

### 2.7. Antibody typing test of bivalent vaccine-immunized mice

SARS-CoV-2 RBD-WT protein was coated on ELISA plates at a concentration of 2 μg/ml and these plates were kept overnight at 4°C. The ELISA plates were washed with PBST twice, and 300 μl of 5% skimmed milk for each well was added, and the plates were incubated at 37°C for 1 h. Serial equal dilutions of mice sera were added, and the plates were incubated at 37°C for 1 h. After the plates were washed four times with PBST, 100 μl/well of IgG1, IgG2a, IgG2b, or IgG3 antibodies were added (1:5,000 dilution), and incubated for 1 h at 37°C. Then the plates were washed four times with PBST and developed with 100 μl/well of TMB one-component chromogenic solution for 5 min and then stopped with 50 μl/well of 2 M H_2_SO_4_. The results were detected with a microplate reader at 450 nm.

### 2.8. ELISpot detection for cellular immunity of bivalent vaccine immunization

In each group, five mice were taken for ELISpot detection. Mouse spleen cells were removed 10 days after the secondary immunization, and ELISpot detection was set up using a negative control group, an experimental group, and a positive control group. The ELISpot plate (Mabtech) was washed with sterile PBS four times, and then blocking buffer (1640 medium +10% FBS + 1% penicillin/streptomycin) was added 200 μl/well and equilibrated for more than 30 min at room temperature. The medium was discarded, and 100 μl of stimulant-free (DMSO containing the same volume as the peptide pool) was added to each well of the negative control group. The SARS-CoV-2 S protein full-length peptide pool (100 μl at 2 μg/ml) was added to each well of the experimental group, and 100 μl of Knife Bean protein A (ConA; 8 μg/ml) was added to each well of the positive control group for assessment of IFN-γ, IL-2, and IL-4 expression. Mouse spleen cells were added at 2 × 10^5^ cells/100 μl, and the plates were placed in 37°C, 5% CO_2_ incubator. After 36 h incubation, the stimulus and cell mixture were discarded, and the plates were washed five times with PBS. Streptavidin-ALP conjugate diluted (1:1,000) with PBS and 0.5% FBS was added and incubated for 1 h at room temperature. Plates were then washed five times with PBS, patted dry, and 100 μl of substrate chromogenic solution (filtered through a 0.45 μm filter) was added to each well for 2–15 min. The bottom of the plate was dried, and fully automated speckle image acquisition and counting was performed using an ELISpot plate reader.

### 2.9. Pseudovirus neutralization assay to detect neutralizing antibody titers

The serum was first diluted with DMEM medium (Gibco, C11995500BT) including 10% fetal bovine serum (FBS; Excell, FND100) and 1% double antibody (WISENT, 450-201-CL), and then filtered through a 0.22 μm filter. Diluted filtered serum (150 μl) was added to first well in a row of an ELISA plate, and then 50 to 100 μl of medium in the next dilution well (three-fold ratio dilution), setting a total of five dilutions (including the first well). Medium was added to the virus control wells (100 μl) and to the cell control wells (150 μl). Pseudoviruses were diluted to 2 × 10^4^ TCID50/mL. Diluted pseudoviruse (50 μl) was added to the sample and virus control wells, shaken and mixed, and then neutralized at 37°C, 5% CO_2_ incubator for 1 h. Digested HEK293-ACE2 cells (Vazyme, DD1401) were diluted to 2 × 10^4^ cells/50 μl and then 50 μl of cell diluent was added per well, shaken and mixed, and incubated for 48 h at 37°C, 5% CO_2_ incubator. Plates were brought to room temperature and the reporter gene assay (Vazyme, DD1201) was added; the fluorescence values were read using an enzyme marker (Tecan, Spark).

### 2.10. Statistical analysis

Data was processed by GraphPad Prism 8.0.1 and statistically analyzed by *t*-test. ns:no significant difference; *p < 0.05; **p < 0.01; ***p < 0.001; ****p < 0.0001.

## 3. Results

### 3.1. Gene cloning, protein purification, and structural analysis of RBD-Beta

**Figure 1:**
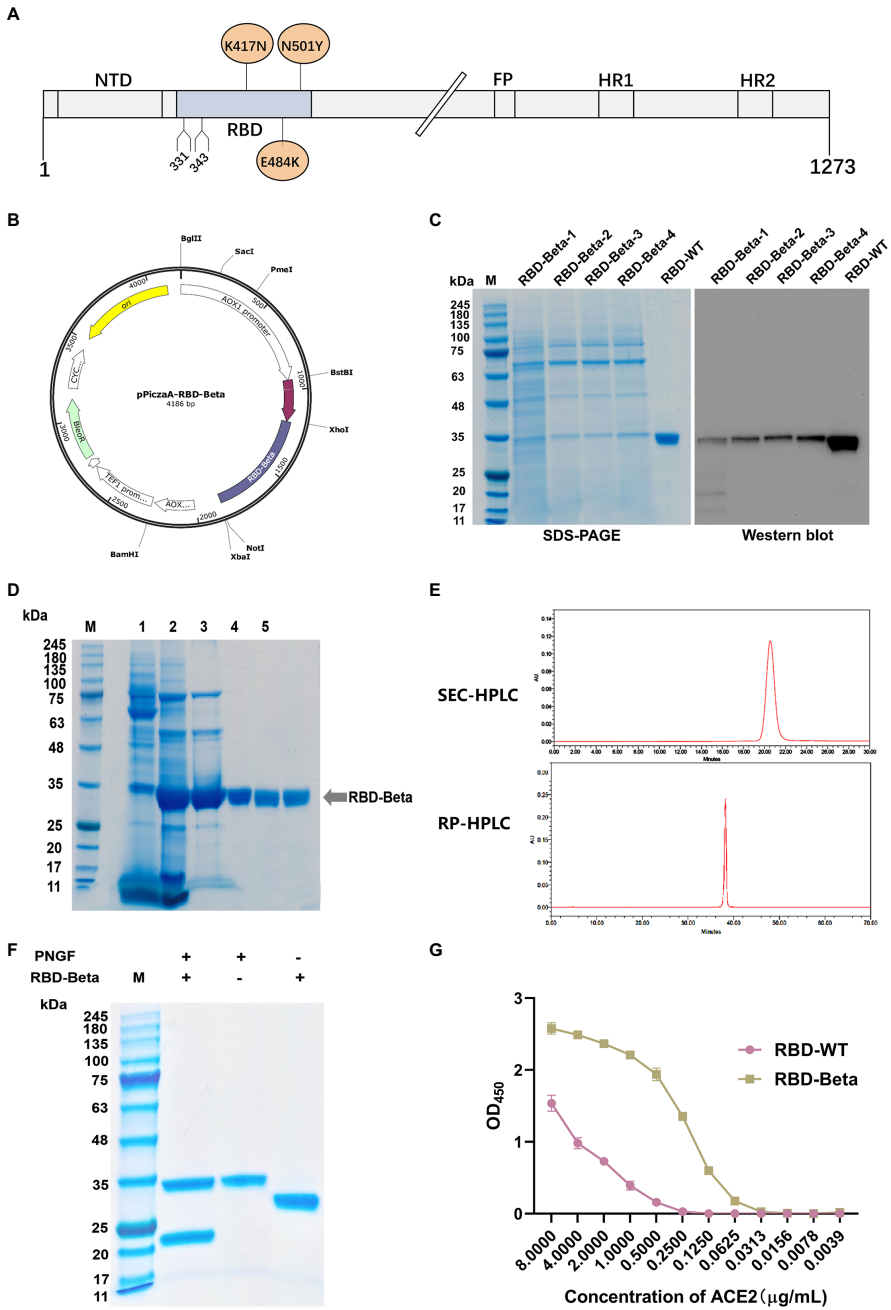
Characterization and expression of the RBD-Beta protein of SARS-CoV-2. **(A)** Schematic sequence of SARS-CoV-2 S protein. **(B)** Map of pPICZαA-RBD-Beta plasmid. **(C)** Analysis of highly expressed clones of RBD-Beta by SDS-PAGE and western blot. **(D)** Analysis of RBD purification process by SDS-PAGE. **(E)** Purity analysis of RBD-Beta protein by SEC-HPLC and RP-HPLC. **(F)** SDS-PAGE results of RBD-Beta digested with PNGF. **(G)** Capacity of RBD-WT and RBD-Beta to bind to human ACE2-His.

The position of SARS-CoV-2 Beta RBD on the S protein is shown in Figure 1A. We successfully cloned the RBD-Beta target gene into the XhoI and NotI sites of pPICZαA (Figure 1B). The positive clone was screened by SDS-PAGE (Figure 1C). The positive strain was expanded, the supernatant was collected and the RBD-Beta protein was purified (Figure 1D). The results of SEC-HPLC and RC-HPLC showed that the purity of the protein was 100% (Figure 1E). Protein digestion *via* PNGF demonstrated that the protein had been glycosylated (Figure 1F). We examined the binding ability of RBDs to hACE2-His by ELISA. This showed that the binding ability of purified glycosylated SARS-CoV-2 Beta RBD to hACE2-His was stronger than that of RBD-WT (Figure 1G), which may be related to the enhanced transmission of the Beta variant.

### 3.2. Sera conjugated and pseudovirus neutralizing antibody titers of RBD-Beta vaccine immunization

**Figure 2:**
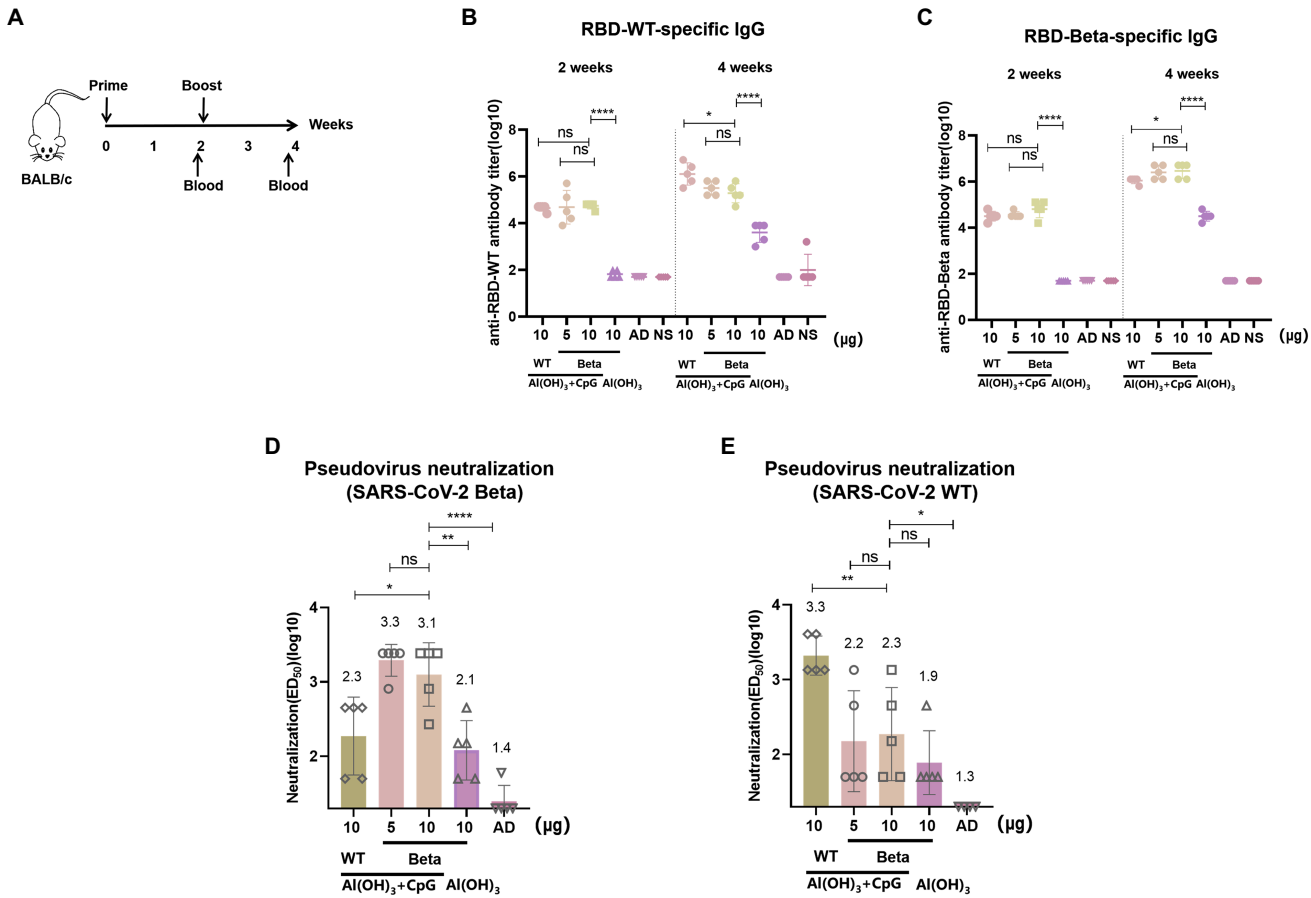
RBD-Beta vaccine immunization BALB/c procedure and antibody titer analysis. **(A)** RBD-Beta vaccine immunization BALB/c mice procedure. **(B)** ELISA analysis of sera conjugated antibody titers for RBD-Beta vaccine immunization of BALB/c mice; the ELISA plates were coated with RBD-WT or RBD-Beta. **(C)** The dotted line divided the RBD-specific antibody titers 2 and 4 weeks after the first immunization. **(D)** Analysis of neutralizing antibody titers of BALB/c sera immunized with RBD-Beta vaccine against SARS-CoV-2 Beta pseudovirus. **(E)** Analysis of neutralizing antibody titers of BALB/c sera immunized with RBD-Beta vaccine against SARS-CoV-2 WT pseudovirus. ns: no significant difference; *p < 0.05; **p < 0.01; ***p < 0.001; ****p < 0.0001.

The immunization schedule of BALB/c mice was shown in Figure 2A. We evaluated RBD-Beta vaccine immune-induced conjugated and neutralizing antibodies in mice sera. Blood was collected from the tail vein of the mice 2 and 4 weeks after the first immunization. The results showed that 2 weeks after the first vaccination there was no significant difference in binding titers between the 10 μg RBD-WT/50 μg CpG/100 μg Al(OH)_3_, 5 μg RBD-Beta/50 μg CpG/100 μg Al(OH)_3_, and 10 μg RBD-Beta/50 μg CpG/100 μg Al(OH)_3_ immunization groups (Figure 2B). There was a significant difference (*p* < 0.0001) in titer between the 10 μg RBD-Beta/50 μg CpG/100 μg Al(OH)_3_ and 10 μg RBD-Beta/100 μg Al(OH)_3_ immunization groups, which indicated that dual adjuvants were necessary. Two weeks after the second vaccination, there was no significant difference in titer between 5 μg RBD-Beta/50 μg CpG/100 μg Al(OH)_3_ and 10 μg RBD-Beta/50 μg CpG/100 μg Al(OH)_3_ immunization groups whether the ELISA plates were coated with RBD-WT or RBD-Beta (Figures 2B,C).

In the pseudovirus neutralization assay, neutralizing antibody titers for SARS-CoV-2 Beta pseudovirus 10 μg RBD-WT/50 μg CpG/100 μg Al(OH)_3_, 5 μg RBD-Beta/50 μg CpG/100 μg Al(OH)_3_, 10 μg RBD-Beta/50 μg CpG/100 μg Al(OH)_3_, and 10 μg RBD-Beta/100 μg Al(OH)_3_ immunization groups were approximately 1:200, 1:1995, 1:1259, and 1:126, respectively (Figure 2D). There was significant difference (*p* = 0.0253) in titer between 10 μg RBD-WT/50 μg CpG/100 μg Al(OH)_3_ and 10 μg RBD-Beta/50 μg CpG/100 μg Al(OH)_3_, and there was significant difference (*p* = 0.0046) in titer between 10 μg RBD-Beta/50 μg CpG/100 μg Al(OH)_3_ and 10 μg RBD-Beta/100 μg Al(OH)_3_ immunization groups. For SARS-CoV-2 WT pseudovirus, the titer of neutralizing antibodies in these groups was approximately 1:1995, 1:158, 1:200, and 1:79, respectively (Figure 2E). Neutralizing antibody titers significantly differed (*p* = 0.0083) between the 10 μg RBD-WT/50 μg CpG/100 μg Al(OH)_3_ and the 10 μg RBD-Beta/50 μg CpG/100 μg Al(OH)_3_ immunization groups.

### 3.3. Sera conjugated antibody titers and antibody typing of bivalent vaccine immunization

**Figure 3:**
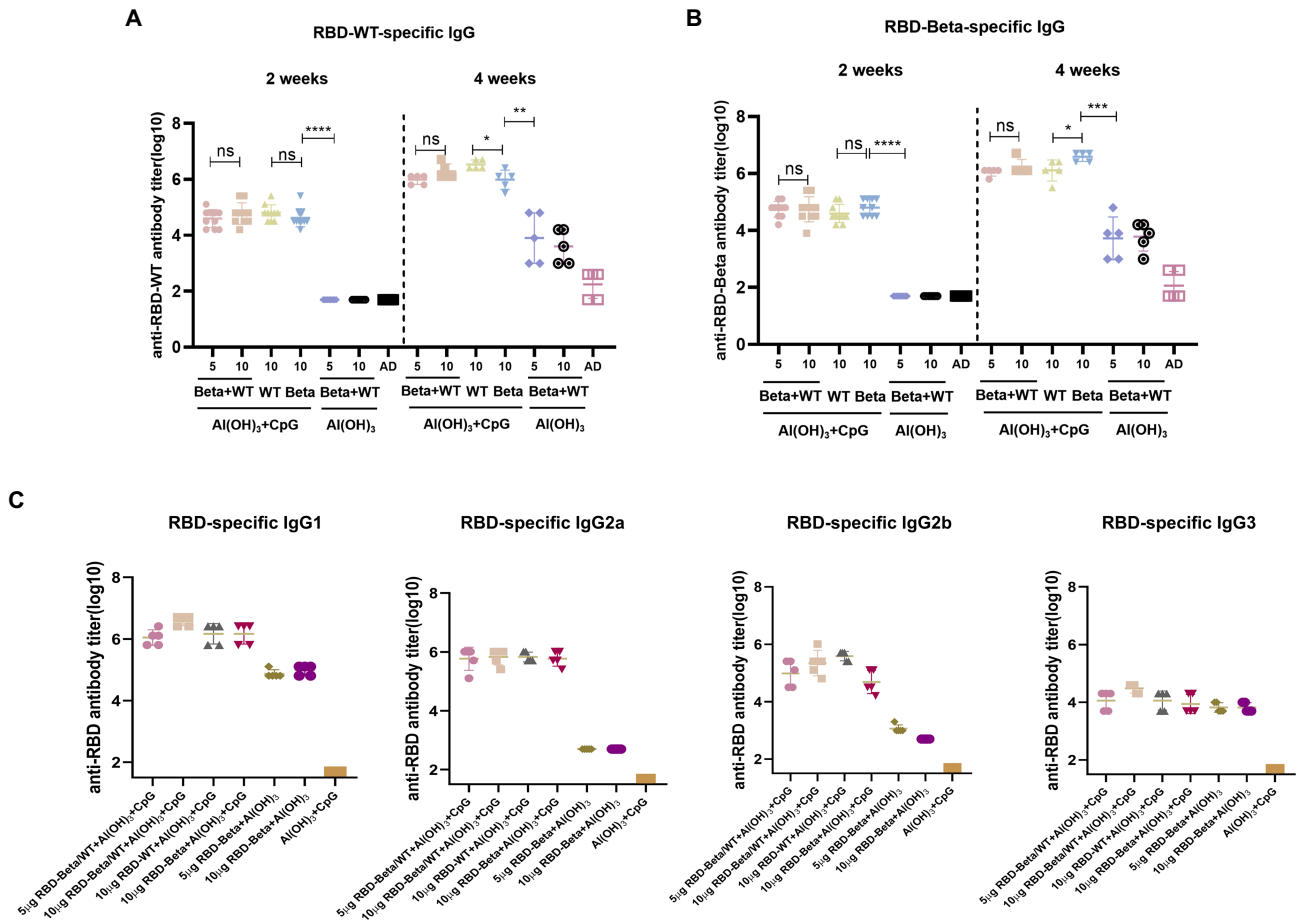
RBD-Beta + RBD-WT bivalent vaccine immunization BALB/c antibody titer detection. **(A)** ELISA assay of RBD-Beta + RBD-WT bivalent vaccine immunization BALB/c sera conjugated antibody titers using RBD-WT and RBD-Beta **(B)** protein-coated plates. The dotted line divided the RBD-specific antibody titers 2 and 4 weeks after the first immunization. **(C)** Sera antibody typing for RBD-Beta + RBD-WT bivalent vaccine immunization of BALB/c mouse. ns: no significant difference; *p < 0.05; **p < 0.01; ***p < 0.001; ****p < 0.0001.

To test for the immunogenicity of the bivalent vaccine, we immunized mice with the bivalent vaccine on days 0 and 14 and performed tail blood sampling for antibody titers 2 weeks after the primary and secondary immunizations, which is the same procedure as shown in Figure 2A. Two weeks after the first immunization, whether vaccinated with RBD-WT, RBD-Beta, or bivalent vaccine with double adjuvants, the titers of all the immunized groups exceeded 10^4^ except for that of the 50 μg CpG/100 μg Al(OH)_3_ immunized group (Figures 3A,B). The bivalent vaccine induced conjugated antibody titers of approximately 10^6^ in mice 2 weeks after the secondary vaccination, and there was no significant difference in the specific antibody titers between the high and low dose groups of the bivalent vaccine (Figure 3A). The titer of the Al(OH)_3_ and CpG double adjuvants groups were significantly higher (*p* = 0.0013, *p* = 0.0002) than that of the Al(OH)_3_ group against RBD-WT and RBD-Beta, respectively (Figures 3A,B). Antibody typing of mice sera showed that the bivalent vaccine elicited robust IgG1, IgG2a, IgG2b, and IgG3 responses (Figure 3C). The specific IgG1, IgG2a, IgG2b, and IgG3 antibody titers induced by 5 μg RBD-WT + 5 μg RBD-Beta/50 μg CpG/100 μg Al(OH)_3_ 2 weeks after the secondary vaccination were over 10^6^, 10^5^, 10^5^, and 10^4^, respectively.

### 3.4. Cytokine assay for bivalent vaccine immunization

**Figure 4:**
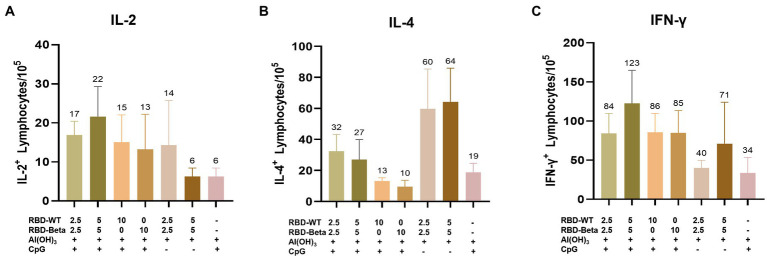
*Ex vivo* ELISpot assay for the IL-2, IL-4, and IFN-γ **(A–C)**. **(A)** IL-2, **(B)** IL-4, and **(C)** IFN-γ expression was detected in mouse splenocytes *via* ELISpot.

To quantify the response of specific T cells to antigenic stimulation, spleen cells from mice 10 days after the second immunization were removed and assayed for IFN-γ and IL-2 secreted by Th1 cells and for IL-4 secreted by Th2 cells using ELISpot. For IL-2 and IFN-γ, the 5 μg RBD-WT + 5 μg RBD-Beta/50 μg CpG/100 μg Al(OH)_3_ group induced 22 spots/10^5^ cells and 123 spots/10^5^ cells, respectively, higher than those of the other groups (Figures 4A,C). The levels of IL-4 were lower in the immune group compared with those in the negative group (Figure 4B), suggesting that the vaccine may have induced cytokine levels more in favor of the Th1 response. Overall, the vaccine induced a higher level of Th1 cellular immune response.

### 3.5. Pseudovirus neutralizing antibody titer test for bivalent vaccine immunization

**Figure 5:**
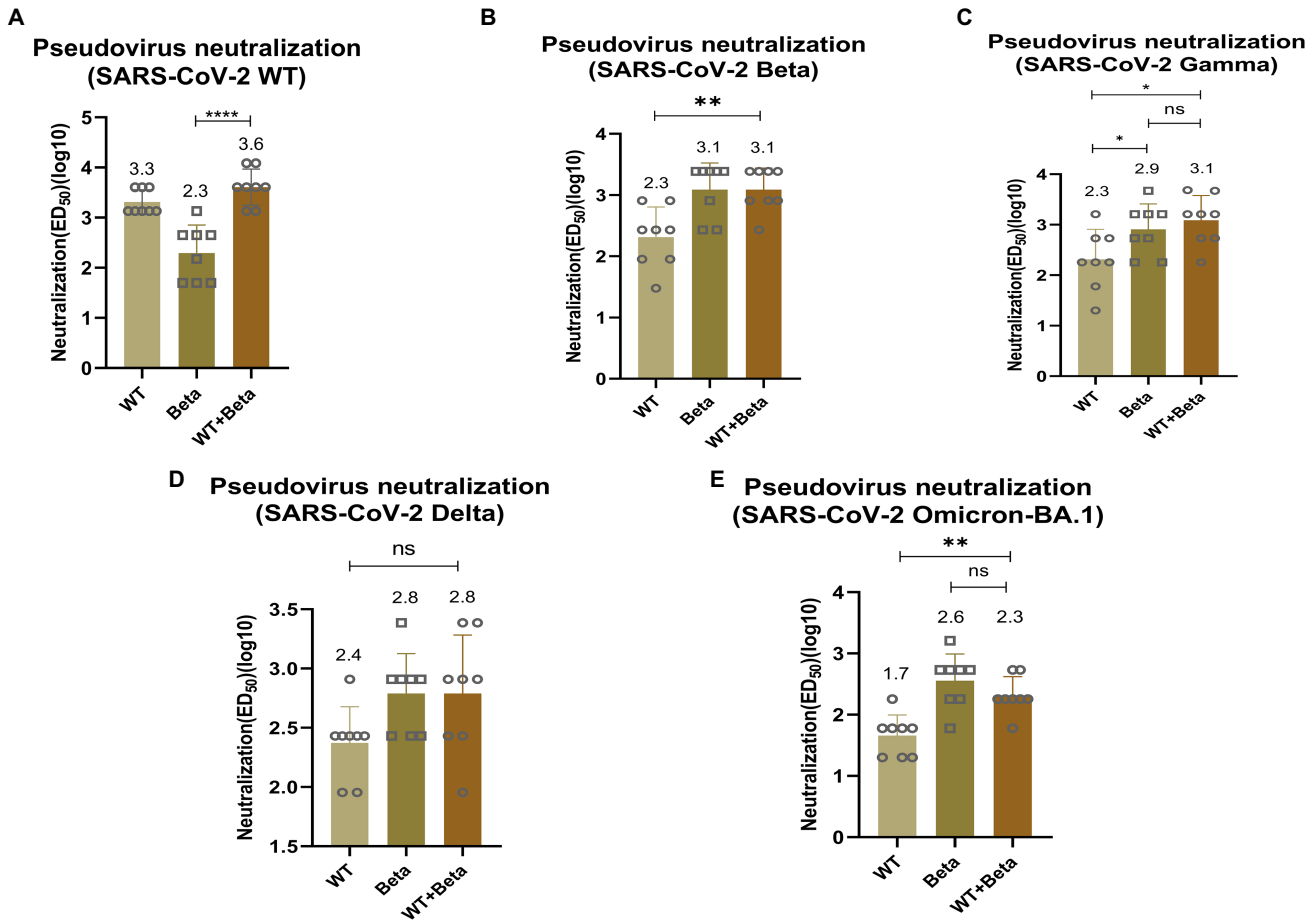
Neutralization activity of BALB/c sera vaccinated by RBD-Beta + RBD-WT bivalent vaccine against SARS-CoV-2 WT **(A)**, Beta **(B)**, Gamma **(C)**, Delta **(D)**, and Omicron **(E)** pseudoviruses. ns: no significant difference; *p < 0.05; **p < 0.01; ***p < 0.001; ****p < 0.0001. ED50: median effective dose.

To detect the efficacy of the bivalent vaccine, mice sera were tested for pseudovirus neutralization titers. For SARS-CoV-2 WT pseudovirus, the bivalent vaccine induced a higher titer of neutralizing antibody (*p* < 0.0001), which was significantly higher than that produced by immunization with RBD-Beta alone (Figure 5A). The titer of neutralizing antibody induced by the bivalent vaccine (*p* = 0.0029) was significantly greater than that produced by immunization with the RBD-WT vaccine for SARS-CoV-2 Beta pseudovirus (Figure 5B). The neutralizing antibody titer induced by the bivalent vaccine was markedly higher than that induced by the monovalent vaccines of RBD-WT (*p* = 0.0132) for SARS-CoV-2 Gamma pseudovirus (Figure 5C). For the Delta pseudovirus, the neutralizing antibody titer induced by the bivalent vaccine was higher than that induced by RBD-WT (Figure 5D). The bivalent vaccine produced higher titers of neutralizing antibody (*p* = 0.0011) against Omicron-BA.1 compared with those produced by the SARS-CoV-2-WT vaccine alone (Figure 5E). However, we found that the neutralizing antibody titers were slightly higher in mice immunized with RBD-Beta alone than in mice immunized with the bivalent vaccine, possibly because of the reduced protective effect of the RBD-WT in the bivalent vaccine against the Omicron. We will subsequently study this issue by setting different vaccine ratios.

## 4. Discussion

The correct glycosylation modification is an important guarantee for immunogenicity. The RBD of SARS-CoV-2 is considered an ideal antigen for vaccine design because of the well-defined structure and mechanism of action and the known major neutralizing epitopes. The RBD acts as the main antigen of SARS-CoV-2 vaccine and induces the immune system to generate specific antibodies against this region, thus providing protection against viral infection. Yeast is a lower eukaryote with the capacity for hypermannosylation, and this modification may mask antigenic epitopes. To address this challenge, we constructed a glycoengineered yeast strain and used yeast glycosylation to resemble the mammalian glycosylation modification pathway. In our previous work, we constructed a glycoengineered yeast positive strain to enable rapid large-scale production of SARS-CoV-2 RBD-WT subunit vaccine (Liu et al., 2022). The RBD-WT subunit vaccine not only showed high purity, but also stimulated robust immune responses in BALB/c mice. After vaccination, the mice showed no adverse conditions such as weight loss, indicating that the vaccine produced by this platform has a certain degree of safety. We produced the RBD-Beta vaccine because of the continuous mutation of SARS-CoV-2, showing that the production process of RBD vaccine can be rapidly scaled up to respond to variants.

Many first-generation vaccines based on the SARS-CoV-2 wild-type strain have been approved or authorized for emergency use, including mRNA, subunit, viral vector, and inactivated vaccines to date (https://www.who.int/). Due to the emergence of novel variants, new vaccine against VOCs should be developed (Garcia-Beltran et al., 2021; Zhou et al., 2021). Therefore, a tailor-made vaccine with broad protective efficacy may provide promising protection against SARS-CoV-2 infection.

The three mutant positions on the RBD of SARS-CoV-2 Beta variant, known as K417N, E484K, and N501Y, reduced vaccine protection for several different technical routes and in COVID-19 donor plasma (Cao et al., 2021; Garcia-Beltran et al., 2021; Wibmer et al., 2021). In the current study, the RBD-Beta monovalent vaccine induced strong neutralizing activity against the Beta variant (Figure 2), and RBD-Beta was consequently considered as an ideal antigen for second-generation vaccine component. The significant decrease in protection against WT pseudovirus by RBD-Beta vaccine suggested that a single variant-based vaccine may not be sufficient to induce cross-neutralizing activity. In addition, the emergence of Omicron and associated subvariants suggests that a broad-spectrum vaccination strategy needs to be explored.

Multivalent vaccines that cover key mutations in the spike protein of SARS-CoV-2 variants may induce a more balanced cross-reactivity. Several studies have shown that bivalent vaccines can induce a broad-spectrum immune response, which would help prevent infections caused by SARS-CoV-2 variants (He et al., 2021; Yuan et al., 2022). Brinkkemper’s study showed that co-display of wild-type and Beta spike proteins of SARS-CoV-2 on self-assembling nanoparticle platform broadens the neutralizing antibody responses (Brinkkemper et al., 2022). Vaccines based on other components, such as S1 or NTD, are also suggested to enhance the broad spectrum of neutralizing antibodies (Jiang et al., 2021; Li et al., 2022; Scheaffer et al., 2022).

Here, the RBD of SARS-CoV-2 Beta variant (RBD-Beta) was expressed successfully, which contains the three mutant sites, K417N, E484K, and N501Y, through a glycoengineered yeast platform. Sera from mice immunized with a bivalent vaccine prepared by mixing RBD-WT and RBD-Beta 1:1 stimulated high titers of conjugated antibodies as well as several types of IgG antibodies (Figure 3). The bivalent vaccine produced higher titers of neutralizing antibodies than when immunized with RBD-WT alone, showing a broad spectrum for neutralizing the mutant pseudoviruses (SARS-CoV-2 Beta, Gamma, Delta, and Omicron pseudoviruses; Figure 5) implying that this may be an effective vaccination strategy to solve the pandemic crisis. Furthermore, the bivalent vaccine induced the secretion of IFN-γ, IL-2, and IL-4 by Th1 and Th2 immune cells, and the bivalent vaccine therefore has the potential to effectively elicit both humoral and cellular responses, which may accelerate the clearance of intracellular microorganisms, such as bacteria and viruses (Figure 4).

Being able to adjust the ratio of bivalent vaccine timely will probably be essential in the future to deal with mutant strains. The recombinant protein-based SARS-CoV-2 vaccine has a promising safety record with no major side effects reported (Keech et al., 2020; Hsieh et al., 2021; Richmond et al., 2021). Vaccine immunogenicity could be improved by adding Al(OH)_3_ and CpG double adjuvants, which was shown to be safe in our previous study (Liu et al., 2022).

There are several limitations to this study. First, we only used the 1:1 vaccine combination, and in response to the continued emergence of SARS-CoV-2 variants, we will continue to investigate the vaccine ratio to find an optimal combination. Second, this study only measured serum conjugated antibodies and neutralizing antibodies at 2 weeks after the secondary vaccination, and continuing monitoring is required to verify the long-term efficacy of the vaccine. Third, the effectiveness of the bivalent vaccine was validated at the mouse level in this study and needs to be validated in other species, such as hamsters and rhesus macaques. Fourth, this study was conducted with pseudovirus only, and to confirm protection in the real world, authentic virus neutralization experiments need to be conducted.

In summary, our data suggested that the bivalent vaccine of SARS-CoV-2 RBD-WT and RBD-Beta could induce broad cross-neutralizing immune responses against SARS-CoV-2 WT, Beta, Gamma, Delta, and Omicron-BA.1 pseudovirus as well as potent T cell responses.

## Data availability statement

The original contributions presented in the study are included in the article/supplementary materials, further inquiries can be directed to the corresponding author.

## Ethics statement

The animal study was reviewed and approved by IACUC-DWZX-2020-039.

## Author contributions

JW and BL: conceptualization, resources, supervision, and project administration. HX and TW: methodology, software, writing—original draft preparation, writing—review and editing, and visualization. HX, TW, and BZ: validation. HX, TW, and PS: formal analysis. HX, TW, and XH: investigation. HX, TW, and XG: data curation. All authors contributed to the article and approved the submitted version.

## Conflict of interest

The authors declare that the research was conducted in the absence of any commercial or financial relationships that could be construed as a potential conflict of interest.

## Publisher’s note

All claims expressed in this article are solely those of the authors and do not necessarily represent those of their affiliated organizations, or those of the publisher, the editors and the reviewers. Any product that may be evaluated in this article, or claim that may be made by its manufacturer, is not guaranteed or endorsed by the publisher.

## Footnotes

^1^ h￼ttps://covid19.who.int/

## References

[ref1] Arunachalam P. S. Walls A. C. Golden N. Atyeo C. Fischinger S. Li C.. (2021). Adjuvanting a subunit COVID-19 vaccine to induce protective immunity. Nature 594, 253–258. doi: 10.1038/s41586-021-03530-2 33873199

[ref2] Brinkkemper M. Veth T. S. Brouwer P. J. M. Turner H. Poniman M. Burger J. A.. (2022). Co-display of diverse spike proteins on nanoparticles broadens sarbecovirus neutralizing antibody responses. iScience 25. doi: 10.1016/j.isci.2022.105649, PMID: 36439375 PMC9678814

[ref3] Cameroni E. Bowen J. E. Rosen L. E. Saliba C. Zepeda S. K. Culap K.. (2022). Broadly neutralizing antibodies overcome SARS-CoV-2 omicron antigenic shift. Nature 602, 664–670. doi: 10.1038/s41586-021-04386-2, PMID: 35016195 PMC9531318

[ref4] Cao Y. Yisimayi A. Bai Y. Huang W. Li X. Zhang Z.. (2021). Humoral immune response to circulating SARS-CoV-2 variants elicited by inactivated and RBD-subunit vaccines. Cell Res. 31, 732–741. doi: 10.1038/s41422-021-00514-9 34021265 PMC8138844

[ref5] Choi B. K. Bobrowicz P. Davidson R. C. Hamilton S. R. Kung D. H. Li H.. (2003). Use of combinatorial genetic libraries to humanize N-linked glycosylation in the yeast Pichia pastoris. Proc. Natl. Acad. Sci. U. S. A. 100, 5022–5027. doi: 10.1073/pnas.0931263100, PMID: 12702754 PMC154291

[ref6] Garcia-Beltran W. F. Lam E. C. St Denis K. Nitido A. D. Garcia Z. H. Hauser B. M.. (2021). Multiple SARS-CoV-2 variants escape neutralization by vaccine-induced humoral immunity. Cells 184, 2372–2383.e2379. doi: 10.1016/j.cell.2021.03.013 PMC7953441 33743213

[ref7] Gstöttner C. Zhang T. Resemann A. Ruben S. Pengelley S. Suckau D.. (2021). Structural and functional characterization of SARS-CoV-2 RBD domains produced in mammalian cells. Anal. Chem. 93, 6839–6847. doi: 10.1021/acs.analchem.1c00893, PMID: 33871970 PMC8078197

[ref8] Harrison A. G. Lin T. Wang P. (2020). Mechanisms of SARS-CoV-2 transmission and pathogenesis. Trends Immunol. 41, 1100–1115. doi: 10.1016/j.it.2020.10.004, PMID: 33132005 PMC7556779

[ref9] Harvey W. T. Carabelli A. M. Jackson B. Gupta R. K. Thomson E. C. Harrison E. M.. (2021). SARS-CoV-2 variants, spike mutations and immune escape. Nat. Rev. Microbiol. 19, 409–424. doi: 10.1038/s41579-021-00573-0 34075212 PMC8167834

[ref10] He C. Yang J. He X. Hong W. Lei H. Chen Z.. (2021). A bivalent recombinant vaccine targeting the S1 protein induces neutralizing antibodies against both SARS-CoV-2 variants and wild-type of the virus. MedComm (2020) 2, 430–441. doi: 10.1002/mco2.72, PMID: 34226895 PMC8242662

[ref11] Hoffmann M. Arora P. Groß R. Seidel A. Hörnich B. F. Hahn A. S.. (2021). SARS-CoV-2 variants B.1.351 and P.1 escape from neutralizing antibodies. Cells 184, 2384–2393.e12. doi: 10.1016/j.cell.2021.03.036, PMID: 33794143 PMC7980144

[ref12] Hsieh S. M. Liu M. C. Chen Y. H. Lee W. S. Hwang S. J. Cheng S. H.. (2021). Safety and immunogenicity of CpG 1018 and aluminium hydroxide-adjuvanted SARS-CoV-2 S-2P protein vaccine MVC-COV1901: interim results of a large-scale, double-blind, randomised, placebo-controlled phase 2 trial in Taiwan. Lancet Respir. Med. 9, 1396–1406. doi: 10.1016/s2213-2600(21)00402-1, PMID: 34655522 PMC8514195

[ref13] Jiang W. Shi L. Cai L. Wang X. Li J. Li H.. (2021). A two-adjuvant multiantigen candidate vaccine induces superior protective immune responses against SARS-CoV-2 challenge. Cell Rep. 37:110112. doi: 10.1016/j.celrep.2021.110112, PMID: 34863353 PMC8610932

[ref14] Keech C. Albert G. Cho I. Robertson A. Reed P. Neal S.. (2020). Phase 1-2 trial of a SARS-CoV-2 recombinant spike protein nanoparticle vaccine. N. Engl. J. Med. 383, 2320–2332. doi: 10.1056/NEJMoa2026920, PMID: 32877576 PMC7494251

[ref15] Kuo T. Y. Lin M. Y. Coffman R. L. Campbell J. D. Traquina P. Lin Y. J.. (2020). Development of CpG-adjuvanted stable prefusion SARS-CoV-2 spike antigen as a subunit vaccine against COVID-19. Sci. Rep. 10:20085. doi: 10.1038/s41598-020-77077-z, PMID: 33208827 PMC7676267

[ref16] Lan J. Ge J. Yu J. Shan S. Zhou H. Fan S.. (2020). Structure of the SARS-CoV-2 spike receptor-binding domain bound to the ACE2 receptor. Nature 581, 215–220. doi: 10.1038/s41586-020-2180-5, PMID: 32225176

[ref17] Li J. Liu Q. Liu J. Fang Z. Luo L. Li S.. (2022). Development of bivalent mRNA vaccines against SARS-CoV-2 variants. Vaccines 10. doi: 10.3390/vaccines10111807, PMID: 36366316 PMC9693459

[ref18] Liu B. Yin Y. Liu Y. Wang T. Sun P. Ou Y.. (2022). A vaccine based on the receptor-binding domain of the spike protein expressed in Glycoengineered Pichia pastoris targeting SARS-CoV-2 stimulates neutralizing and protective antibody responses. Engineering 13, 107–115. doi: 10.1016/j.eng.2021.06.012, PMID: 34457370 PMC8378774

[ref19] Moyo-Gwete T. Madzivhandila M. Makhado Z. Ayres F. Mhlanga D. Oosthuysen B.. (2021). Cross-reactive neutralizing antibody responses elicited by SARS-CoV-2 501Y.V2 (B.1.351). N. Engl. J. Med. 384, 2161–2163. doi: 10.1056/NEJMc2104192, PMID: 33826816 PMC8063886

[ref20] Planas D. Saunders N. Maes P. Guivel-Benhassine F. Planchais C. Buchrieser J.. (2022). Considerable escape of SARS-CoV-2 omicron to antibody neutralization. Nature 602, 671–675. doi: 10.1038/s41586-021-04389-z, PMID: 35016199

[ref21] Richmond P. Hatchuel L. Dong M. Ma B. Hu B. Smolenov I.. (2021). Safety and immunogenicity of S-trimer (SCB-2019), a protein subunit vaccine candidate for COVID-19 in healthy adults: a phase 1, randomised, double-blind, placebo-controlled trial. Lancet 397, 682–694. doi: 10.1016/s0140-6736(21)00241-5, PMID: 33524311 PMC7906655

[ref22] Scheaffer S. M. Lee D. Whitener B. Ying B. Wu K. Liang C. Y.. (2022). Bivalent SARS-CoV-2 mRNA vaccines increase breadth of neutralization and protect against the BA.5 omicron variant in mice. Nat. Med. doi: 10.1038/s41591-022-02092-8, PMID: 36265510 PMC11752949

[ref23] Souza P. F. N. Mesquita F. P. Amaral J. L. Landim P. G. C. Lima K. R. P. Costa M. B.. (2022). The spike glycoprotein of SARS-CoV-2: a review of how mutations of spike glycoproteins have driven the emergence of variants with high transmissibility and immune escape. Int. J. Biol. Macromol. 208, 105–125. doi: 10.1016/j.ijbiomac.2022.03.058, PMID: 35300999 PMC8920968

[ref24] Tegally H. Wilkinson E. Giovanetti M. Iranzadeh A. Fonseca V. Giandhari J.. (2021). Detection of a SARS-CoV-2 variant of concern in South Africa. Nature 592, 438–443. doi: 10.1038/s41586-021-03402-9 33690265

[ref25] Tuekprakhon A. Nutalai R. Dijokaite-Guraliuc A. Zhou D. Ginn H. M. Selvaraj M.. (2022). Antibody escape of SARS-CoV-2 omicron BA.4 and BA.5 from vaccine and BA.1 serum. Cells 185, 2422–2433.e13. doi: 10.1016/j.cell.2022.06.005, PMID: 35772405 PMC9181312

[ref26] Wang G. L. Wang Z. Y. Duan L. J. Meng Q. C. Jiang M. D. Cao J.. (2021). Susceptibility of circulating SARS-CoV-2 variants to neutralization. N. Engl. J. Med. 384, 2354–2356. doi: 10.1056/NEJMc2103022, PMID: 33822491 PMC8063885

[ref27] Wibmer C. K. Ayres F. Hermanus T. Madzivhandila M. Kgagudi P. Oosthuysen B.. (2021). SARS-CoV-2 501Y.V2 escapes neutralization by south African COVID-19 donor plasma. Nat. Med. 27, 622–625. doi: 10.1038/s41591-021-01285-x, PMID: 33654292

[ref28] Yuan M. Huang D. Lee C. D. Wu N. C. Jackson A. M. Zhu X.. (2021). Structural and functional ramifications of antigenic drift in recent SARS-CoV-2 variants. bioRxiv. doi: 10.1101/2021.02.16.430500, PMID: 34016740 PMC8284396

[ref29] Yuan Y. Zhang X. Chen R. Li Y. Wu B. Li R.. (2022). A bivalent nanoparticle vaccine exhibits potent cross-protection against the variants of SARS-CoV-2. Cell Rep. 38:110256. doi: 10.1016/j.celrep.2021.110256, PMID: 34990583 PMC8695190

[ref30] Zhang L. Li Q. Liang Z. Li T. Liu S. Cui Q.. (2022). The significant immune escape of pseudotyped SARS-CoV-2 variant omicron. Emerg. Microbes Infect. 11, 1–5. doi: 10.1080/22221751.2021.2017757, PMID: 34890524 PMC8725892

[ref31] Zhou D. Dejnirattisai W. Supasa P. Liu C. Mentzer A. J. Ginn H. M.. (2021). Evidence of escape of SARS-CoV-2 variant B.1.351 from natural and vaccine-induced sera. Cells 184, 2348–2361.e6. doi: 10.1016/j.cell.2021.02.037, PMID: 33730597 PMC7901269

